# Heterozygosity of lysosomal enzyme genes and Alzheimer's disease

**DOI:** 10.1002/alz70855_101638

**Published:** 2025-12-23

**Authors:** Mark Sands, Bruno A. Benitez, Clare E Wallace, Maulikkumar P Patel, Niko‐Petteri Nykanen, Carla M Yuede, Samantha L Eaton, Cyril P Pottier, Arda Cetin, Matthew Johnson, Mia Bevan, Woodrow Gardiner, Hannah M Edwards, Brookelyn Doherty, Ryan Harrigan, Dominic Kurian, Thomas M Wishart, Colin Smith, Neal Goodwin, John R Cirrito

**Affiliations:** ^1^ Washington University School of Medicine, Elberton, GA, USA; ^2^ Beth Israel Deaconess Medical Center, Boston, MA, USA; ^3^ Washington University School of Medicine, Saint Louis, MO, USA; ^4^ Department of Psychiatry, Washington University School of Medicine, St. Louis, MO, USA; ^5^ The University of Edinburgh, Edinburgh, United Kingdom; ^6^ Department of Psychiatry, Washington University, Saint Louis, MO, USA; ^7^ Washington University School of Medicine, St. Louis, MO, USA; ^8^ Roslin Institute, Edinburgh, EH259RG, United Kingdom; ^9^ Univeristy of Edinburgh, Edinburgh, United Kingdom; ^10^ Jaya Biosciences, San Francisco, CA, USA

## Abstract

**Background:**

Although lysosome dysfunction has been implicated in Alzheimer's disease (AD), it is unclear what level or type of dysfunction is pathogenic. We hypothesize that haploinsufficiency of lysosomal enzymes is associated with AD and that CNS‐directed, AAV‐mediated gene transfer can be an effective treatment.

**Method:**

Although lysosome dysfunction has been implicated in Alzheimer's disease (AD), it is unclear what level or type of dysfunction is pathogenic. We hypothesize that haploinsufficiency of lysosomal enzymes is associated with AD and that CNS‐directed, AAV‐mediated gene transfer can be an effective treatment.

**Result:**

Heterozygous protein‐damaging mutations in several lysosomal enzyme genes are enriched in AD patients compared to matched controls. Proteomic analysis shows that the lysosomal storage disease pathway is activated in the brains of AD patients. There is a gene‐dosage effect on Ab_40_ levels in brain ISF between WT, heterozygous, and homozygous *PPT1* deficient mice. Although APP is not changed, the levels of a‐, b‐, and g‐secretases are altered in *PPT1* heterozygous mice in a pattern that favors an amyloidogenic pathway. Heterozygosity of *PPT1* increases Ab plaques, insoluble Ab40 and Ab42 levels, and decreases the life span of 5xFAD mice. Consistent with the human genetic and murine data, the AD pathway is activated in heterozygous *PPT1* sheep. AAV‐mediated gene therapy in 5xFAD/PPT1+/‐ mice decreased the Ab burden, increased life span, and improved cognitive function. The effects of heterozygosity of the *NAGLU, GALC, IDUA*, and *GUSB* genes on Ab pathology were nearly identical to those observed for *PPT1* heterozygosity.

**Conclusion:**

These data strongly implicate heterozygosity of at least five, likely more, lysosomal enzyme genes in the development of AD and these genes might be effective therapeutic targets in certain genetically‐defined forms of AD.

